# Thyroid Allostasis–Adaptive Responses of Thyrotropic Feedback Control to Conditions of Strain, Stress, and Developmental Programming

**DOI:** 10.3389/fendo.2017.00163

**Published:** 2017-07-20

**Authors:** Apostolos Chatzitomaris, Rudolf Hoermann, John E. Midgley, Steffen Hering, Aline Urban, Barbara Dietrich, Assjana Abood, Harald H. Klein, Johannes W. Dietrich

**Affiliations:** ^1^Medical Department I, Endocrinology and Diabetology, Bergmannsheil University Hospitals, Ruhr University of Bochum, Bochum, Germany; ^2^Private Consultancy, Research and Development, Yandina, QLD, Australia; ^3^North Lakes Clinical, Ilkley, United Kingdom; ^4^Department for Internal Medicine, Cardiology, Endocrinology, Diabetes and Medical Intensive Care Medicine, Krankenhaus Bietigheim-Vaihingen, Bietigheim-Bissingen, Germany; ^5^Department for Anesthesiology, Intensive Care and Palliative Medicine, Eastern Allgäu-Kaufbeuren Hospitals, Kaufbeuren, Germany; ^6^kbo-Isar-Amper-Klinikum, Klinikum München-Ost, Haar, Germany; ^7^Ruhr Center for Rare Diseases (CeSER), Ruhr University of Bochum and Witten/Herdecke University, Bochum, Germany

**Keywords:** thyroid allostasis, non-thyroidal illness syndrome, thyroid hormone metabolism, hypothalamus–pituitary–thyroid feedback control, TACITUS syndrome

## Abstract

The hypothalamus–pituitary–thyroid feedback control is a dynamic, adaptive system. In situations of illness and deprivation of energy representing type 1 allostasis, the stress response operates to alter both its set point and peripheral transfer parameters. In contrast, type 2 allostatic load, typically effective in psychosocial stress, pregnancy, metabolic syndrome, and adaptation to cold, produces a nearly opposite phenotype of predictive plasticity. The non-thyroidal illness syndrome (NTIS) or thyroid allostasis in critical illness, tumors, uremia, and starvation (TACITUS), commonly observed in hospitalized patients, displays a historically well-studied pattern of allostatic thyroid response. This is characterized by decreased total and free thyroid hormone concentrations and varying levels of thyroid-stimulating hormone (TSH) ranging from decreased (in severe cases) to normal or even elevated (mainly in the recovery phase) TSH concentrations. An acute versus chronic stage (wasting syndrome) of TACITUS can be discerned. The two types differ in molecular mechanisms and prognosis. The acute adaptation of thyroid hormone metabolism to critical illness may prove beneficial to the organism, whereas the far more complex molecular alterations associated with chronic illness frequently lead to allostatic overload. The latter is associated with poor outcome, independently of the underlying disease. Adaptive responses of thyroid homeostasis extend to alterations in thyroid hormone concentrations during fetal life, periods of weight gain or loss, thermoregulation, physical exercise, and psychiatric diseases. The various forms of thyroid allostasis pose serious problems in differential diagnosis of thyroid disease. This review article provides an overview of physiological mechanisms as well as major diagnostic and therapeutic implications of thyroid allostasis under a variety of developmental and straining conditions.

## Introduction

Contemporary diagnosis of thyroid disorders relies predominantly on point measurements of thyrotropin [thyroid-stimulating hormone (TSH)] concentration (1). While some guidelines recommend combining TSH measurements with free thyroxine (FT4) determination (2, 3), others constrain diagnostic workup on TSH measurements as a first-line diagnostic test and only recommend determining peripheral thyroid hormones, if TSH concentrations fall outside of their respective reference ranges (4–6). This strategy rests upon the assumptions of a log-linear relationship between TSH and FT4 (7–10), a long plasma half-life of thyroid hormones (11, 12) and tight coupling of all involved control elements of the feedback loop (13, 14). While TSH-based diagnostic interpretation may be inexpensive (at least at the beginning of the decision-making process) it is over-simplifying and involves considerable risks of both false positive and false negative results. Restrictions of TSH-based protocols include circadian and ultradian variation of TSH and thyroid hormones (15–18), the plasticity of central components of the feedback loop under substitution therapy with levothyroxine (19–22) and, as we will subsequently outline, reactive adjustments of thyroid homeostasis in certain phases of development and conditions of strain and stress (23–29).

**Figure 1:**
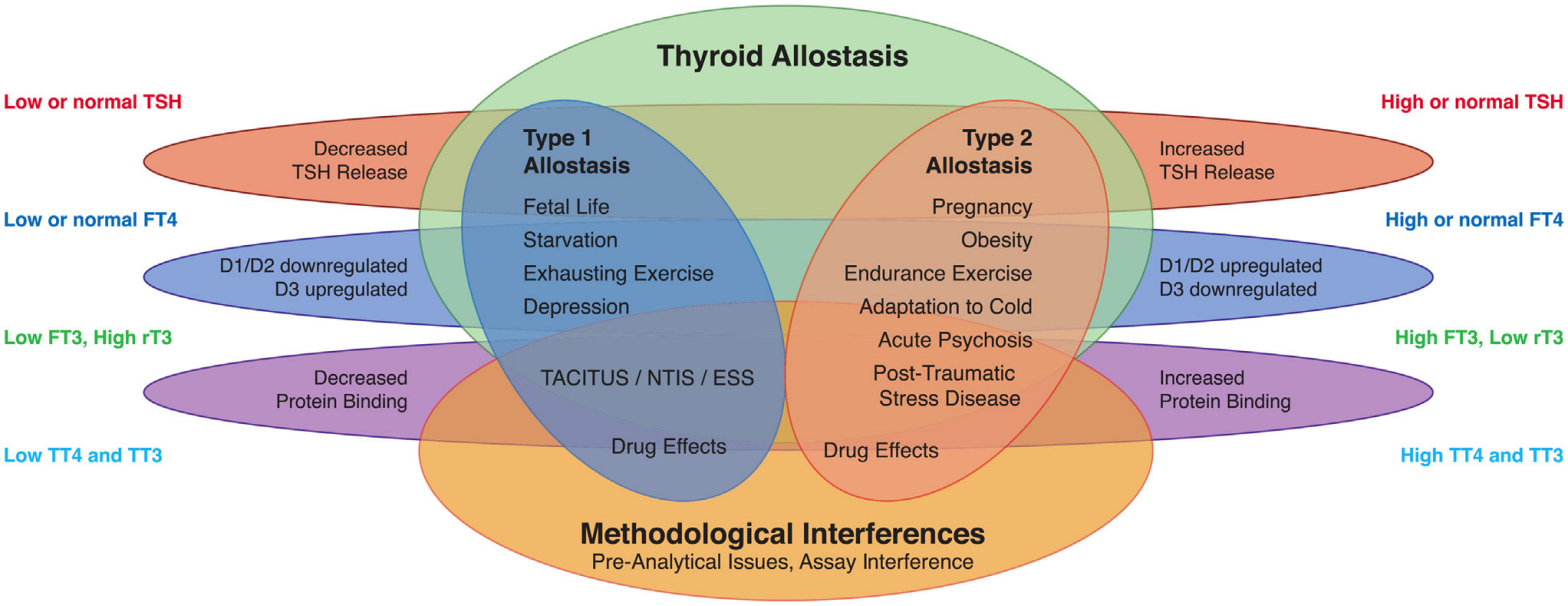
Altered concentrations of thyroid hormones in certain life situations may result from type 1 allostatic load (comprising thyrotropic adaptation, hypodeiodination, and decreased protein binding of thyroid hormones), type 2 allostatic load [showing increased thyroid-stimulating hormone (TSH) release, hyperdeiodination, and augmented binding of thyroid hormones to plasma proteins], and non-homeostatic mechanisms including methodological interferences (53).

The hypothalamic-pituitary-thyroid (HPT) axis acts as an adaptive, dynamic system, functioning in two distinct operating modes. The system operates as a homeostatic regulator in unstrained resting conditions, aiming at constant value control (30–33) and maintaining serum concentrations of thyroid hormones in the vicinity of a fixed set point (17, 18, 34–38). The stable situation in equilibrium permits the use of TSH measurement for diagnostic purposes in thyroid disease. However, concentrations of TSH and thyroid hormones may be altered in other physiological and pathological situations in the absence of any dysfunction of the thyrotropic control system or any of its elements (23–25, 39–42). The feedback control mechanism is able to modify its transfer parameters, if a need arises, to tune consumption to available supply with oxygen, energy, and glutathione. The operating mode then shifts to a system of tracking control, which features a dynamically changing set point (26, 43–45). Clinical patterns emerging from this kind of adaptation are well known to physicians. They typically include, but are not limited to, altered, either low or high, T3 concentrations, changes in binding of thyroid hormones to plasma proteins and adjustment of the central control input. In humans, allostatic operation of the HPT axis was initially described in exhausting exercise, starvation, and systemic illness (46–52). Similar patterns were later observed under such diverse conditions as fetal life, major depression (MD), and space flight. However, adding further to the complexity of the constellation, opposite changes have been described in other situations such as pregnancy, endurance exercise, and certain psychiatric diseases (Figure 1) (27–29).

The characteristic adaptive constellation of thyroid homeostasis to severe illness is referred to as low-T3 syndrome, non-thyroidal illness syndrome (NTIS), euthyroid sick syndrome (ESS) or thyroid allostasis in critical illness, tumors, uremia, and starvation (TACITUS). About 30% of hospitalized patients (54) and more than 60% of patients affected by critical illness (55, 56) experience transient changes in serum concentrations of TSH and thyroid hormones. Characteristic patterns are low levels of free and total 3,3′,5-triiodothyronine (T3) (39, 40, 56), impaired plasma protein binding of thyroid hormones (57, 58) and, in more severe cases, thyrotropic adaptation with a downward shift of the set point characterized by paradoxically low TSH levels in the presence of normal or even low concentrations of FT4 (59, 60). Conversely, serum concentrations of 3,5,5′-triiodothyronine (rT3) and 3,5-diiodothyronine (3,5-T2) are typically increased (39, 40, 61–63).

In severe illness, the presence of NTIS predicts poor prognosis (54, 60, 64–66). It is still a matter of fierce debate if patients affected by the syndrome may benefit from substituting thyroid hormones (67–70). Importantly, significant problems in differential diagnosis may arise from both considerable overlap of hormone concentrations in NTIS with those in primary or secondary thyroid disorders and by methodological interference with thyroid hormone assays (71–73).

This review article provides an overview of adaptive responses of thyroid homeostasis in type 1 and type 2 allostatic situations. It is based on a broad literature search executed with the search formulas “non-thyroidal illness OR non-thyroidal illness OR NTI OR NTIS OR TACITUS OR euthyroid sick OR low T3 OR low triiodothyronine OR Euthyroid Sick Syndromes [MeSH],” “(thyroid OR thyroxine OR triiodothyronine) AND (allostasis OR allostatic)” and “amygdala AND TRH” in PubMed, the authors’ own collections of literature and secondary publications referenced there. Where not otherwise specified information provided refers to the human organism. Data from animal research are reported, where information on the human metabolism is lacking.

## Historical Overview

Perhaps the first description of NTIS dates back to the tenth century BC, when King David was on his deathbed: “*Now King David was old, and advanced in years: and when he was covered with clothes he was not warm*.” [1 Kings 1:1]. Of note, David was not mentally impaired, since in the same time he had managed to defend Solomon, his designated successor, against a subtle conspiracy. Therefore, the situation described by the unknown author seems to represent an exclusively peripheral reduction of thyroid hormones, perhaps in the context of senescence and/or multi-morbidity.

In the human organism, transient alterations of thyroid hormone metabolism unrelated to pituitary or thyroid disease were first explicitly described in 1968, when Clifford Irvine reported reduced half-life of T4 in athletic training, which was reversible after three days’ rest (74). The same author had made similar observations in horses, where thyroxine secretion rate increased in training and adaptation to cold, and half-life decreased in trained animals (75). Shortly later, Harland and Orr described a significantly decreased half-life of T4, when rats were exposed to a cold environment (76). Transiently changed concentrations of thyroid hormones were first described in 1971 by Terjung and Tipton, who reported increased concentrations of free and total T4 during bicycle ergometer training and reduced total T4 levels 24 h later (46).

In 1973, Rothenbuchner et al. reported decreased serum concentrations of T3 in the starving organism (47). Nearly simultaneously another group confirmed this observation in a different population (49). Shortly thereafter T3 concentrations were observed to be reduced in patients with critical illness requiring intensive care, in tumors and in uremia (48, 51, 52, 77).

The last four decades witnessed the discovery of many more pathologies that are associated with the low-T3 syndrome or other patterns of NTIS, including sepsis, circulatory arrest, stroke, myocardial infarction, pulmonary embolism, inflammatory bowel disease, renal failure, and gastrointestinal fistulae (24, 25, 39, 56, 66).

A pattern typical of “NTIS” was also observed specifically in non-pathological conditions, such as the fetal period, torpor in poikilotherm animals, and hibernation in certain mammalian species (78, 79). These observations suggested that ESS is not a dysfunction of the feedback loop, rather an allostatic reaction and potentially useful adaptation of the pituitary–thyroid feedback control system to reduced supplies in energy, oxygen, and glutathione. We therefore recently coined a new term of TACITUS to provide a more neutral designation that encompasses several non-pathological conditions with adaptations of TSH and thyroid hormones (18, 35).

## Mechanisms of Thyroid Allostasis

In situations of current or anticipated strain central and peripheral mechanisms interact to ensure a coordinated adaptation of thyroid hormone signaling (43). This is associated with a variety of alterations at the molecular level in nearly all tissues.

### Cybernetic Principles of Integrative Thyroid Control

**Figure 2:**
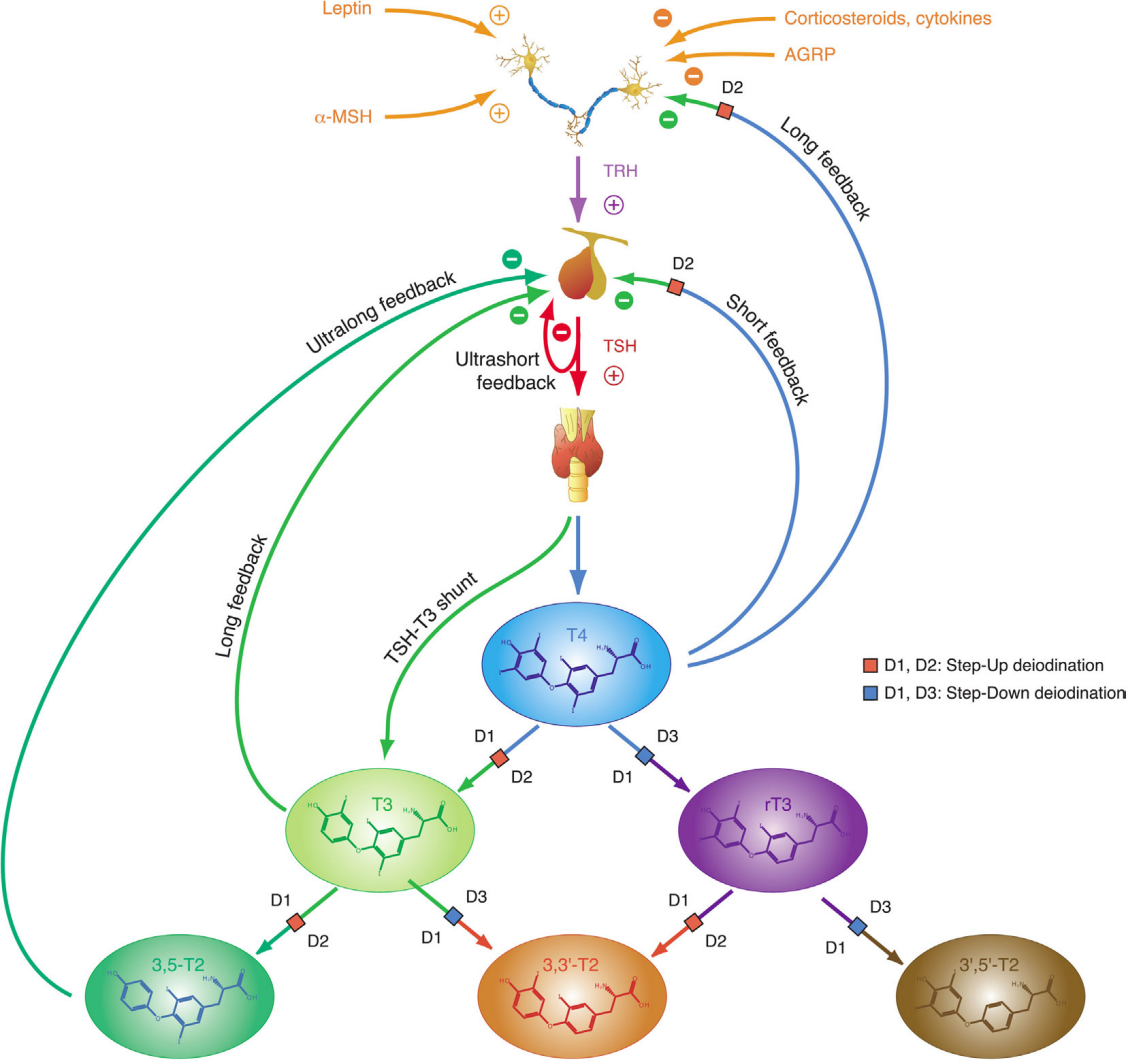
Thyroid homeostasis comprises ultrashort, short, and long feedback mechanisms. In addition, conversion between iodothyronines is adaptively mediated by three distinct deiodinases (18, 35). Deiodination is controlled by multiple local and global mediators including thyroid-stimulating hormone (TSH).

Homeostatic control of thyroid function represents a classical example of a hypothalamic–pituitary-mediated endocrine feedback mechanism (Figure 2) (18, 35). Its principal mediators are hypothalamic thyrotropin-releasing hormone (TRH), pituitary thyrotropin (TSH), thyroxine (T4), and triiodothyronine (T3).

Thyroid-stimulating hormone is a glycoprotein hormone with a rather short half-life of 50–60 min, which stimulates synthesis and release of T4 and to a lesser degree T3 from the thyroid gland *via* binding to a specific TSH receptor (35). With respect to classical thyroid hormone actions (effects mediated *via* nuclear thyroid hormone receptors) T4 is a prohormone requiring activation to the highly agonistic hormones T3 and 3,5-T2 to be effective. However, other non-classical actions of T4, acting, e.g., *via* integrin receptors, do not require prior activation, rendering T4 an active hormone with respect to non-classical effects (80).

Plasma T3 is derived from several sources, including direct formation in the thyroid gland and release by proteolysis of thyroglobulin (Tg), deiodination from T4 in the thyroid and deiodination in peripheral organs (12). T4 and T3 display prolonged plasma half-lives of 1 week and 1 day, respectively, resulting from intracellular accumulation and a high proportion of plasma protein binding (17, 35, 81). T3 affects most tissues in a pleiotropic manner. It also closes the feedback loop by inhibiting both synthesis and release of TSH from the pituitary gland (17, 18, 35).

This “short” feedback loop is augmented by additional control motifs, including an ultrashort feedback loop of TSH release (17, 82), a long feedback loop, where thyroid hormones inhibit TRH release in the hypothalamus (83, 84), and a direct stimulatory effect of TSH on T3 formation. A TSH–T3 shunt has been predicted in animal and cell culture experiments (85–90), and we recently demonstrated its existence in the human organism (19, 22, 91–94).

**Figure 3:**
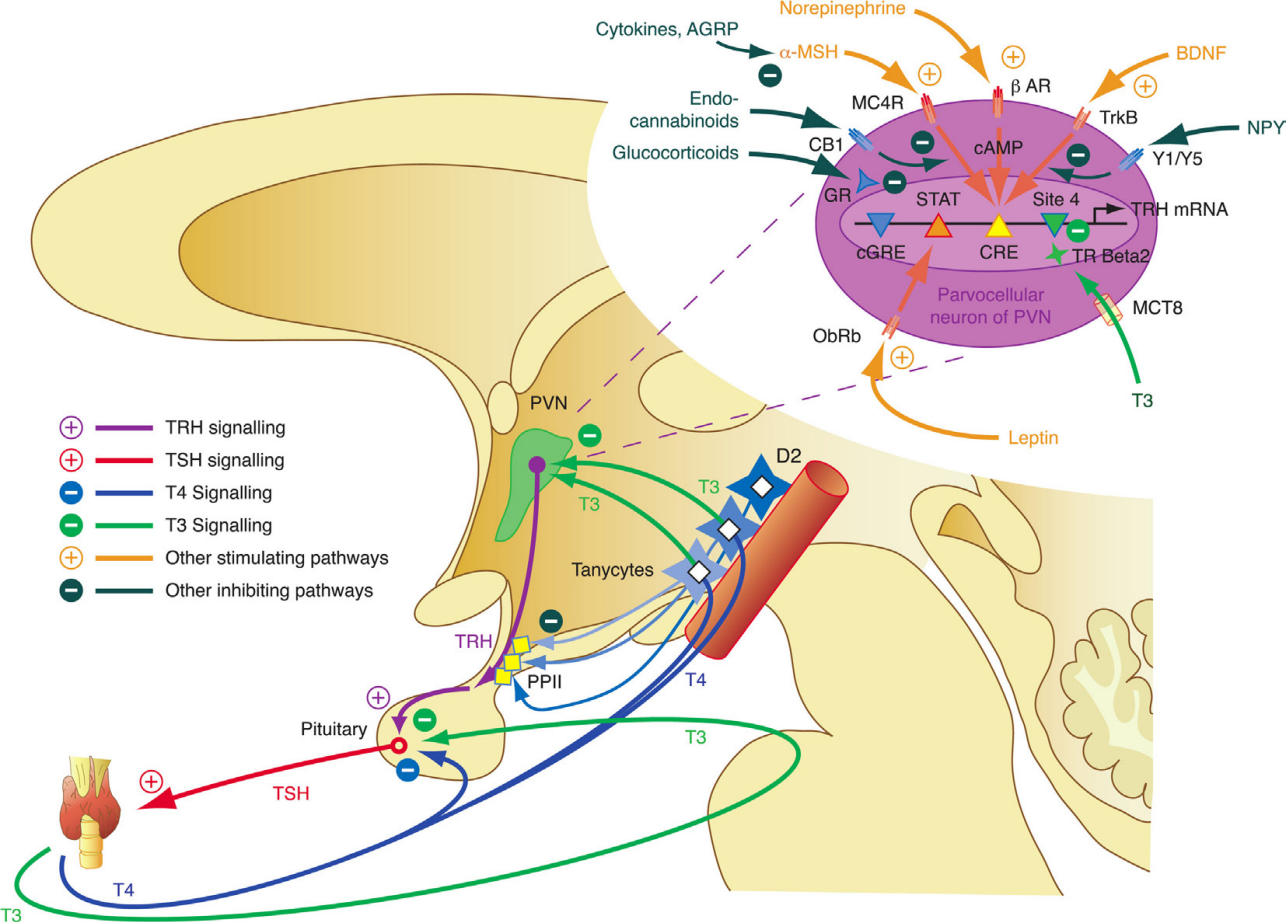
Critical components of the central governor of thyroid homeostasis include parvocellular thyrotropin-releasing hormone (TRH) neurons, which integrate multiple afferent signals relaying information on nutrition and stress, and tanycytes lining the third ventricle at the blood–brain barrier, which are able to control both synthesis and degradation of thyrotropin-releasing hormone (TRH) *via* type 2 deiodinase (D2) and pyroglutamyl peptidase II (PPII) (95, 97–99).

The integrative control at the hypothalamic level is mediated by parvocellular hypophysiotropic paraventricular nucleus (PVN) TRH neurons. Their activity provides an interface between thyroid hormone feedback, nutritional status, and stimulatory or inhibitory influences of the circadian rhythms (Figure 3) (95). In this respect, tanycytes lining the third ventricle play a pivotal role in central homeostasis. They are able to fine-tune the sensitivity of PVN *via* provision of central T3 and to degrade TRH *via* pyroglutamyl peptidase II (PPII) at the level of the median eminence (Figure 3) (95, 96).

### Allostasis and Allostatic Load

**Figure 4:**
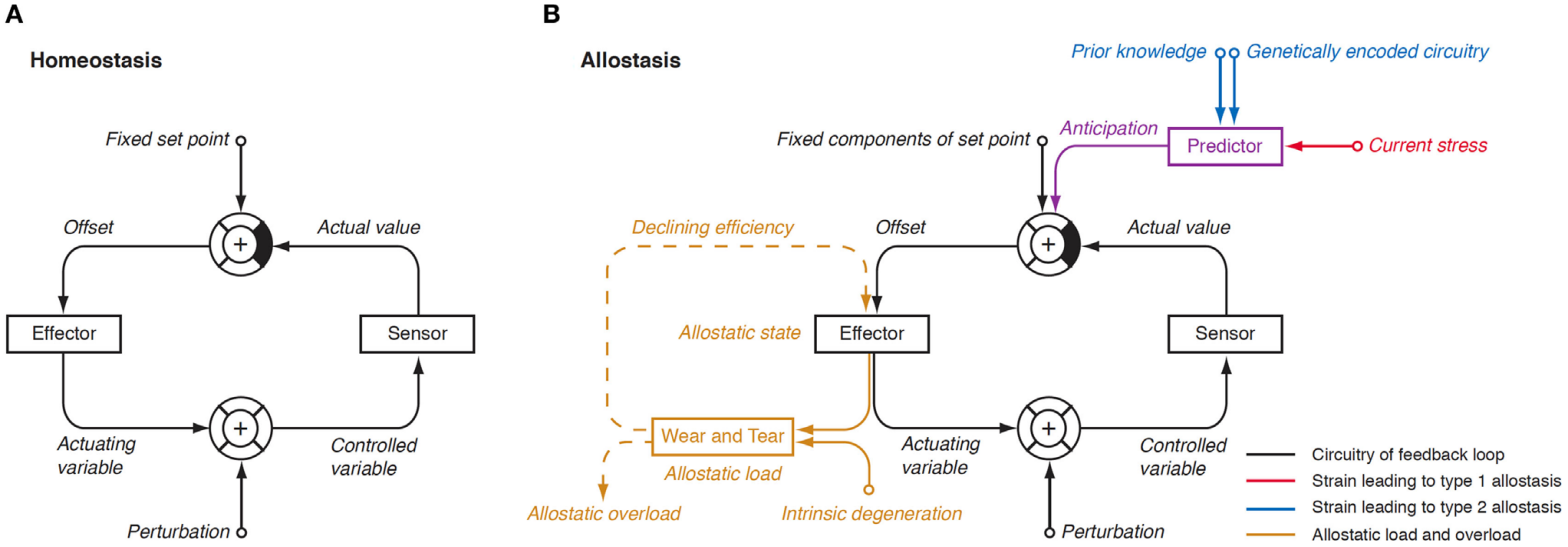
**(A)** In a physiological homeostatic system, afferent information is compared with a fixed set point, and the sensed discrepancy leads to counter-regulatory activity of the effector. Negative (degenerative) feedback ensures static stability of the system. **(B)** In situations of allostasis, stress signals are looped in at a central level, thus resulting in ongoing offset signaling. Due to saturation of receptors and enzymes, this discrepancy reduces the efficiency of the effector and, if ongoing, gives rise to “wear and tear” reactions. Both mechanisms combine as allostatic load, which may be a source of pathology on its own (105, 106).

In 1988, Sterling and Eyer extended the classical paradigm of homeostasis with the theory of allostasis (100). Briefly, an allostatic response is defined as a dynamic stress reaction that maintains stability through change (101). This distinct operating mode of homeostatic systems becomes apparent in straining and occasionally life-threatening situations. Allostasis both contains and extends the homeostatic principles by adapting set points and other boundaries of control (Table 1) (101). The primary mediators of allostatic response include but are not limited to catecholamines, hormones of the hypothalamo-pituitary–adrenal (HPA) axis and cytokines (102). Of note, allostasis deals with a trade-off situation. It ensures survival in extreme situations, where, e.g., the demand of energy exceeds supply, but this protective reaction occurs at the expenses of a stress reaction (referred to as *allostatic state*), which, in turn, may have adverse consequences of its own. The cumulative result of an allostatic state is referred to as *allostatic load* of the organism (Figure 4). If allostatic load remains excessive or persists over a period of time it may confer pathology and turn out to be life threatening by its own nature (*allostatic overload*).

**Table 1:** Key concepts of homeostasis and allostasis (103, 104).

Homeostasis	Allostasis
Constant or oscillating set point	Changing set point
Physiologic equilibrium	Compensated equilibrium
No or little anticipation^a^ of demand	Extensive anticipation of demand
No adjustment based on history	Adjustment based on history
Adjustment carries no price	Adjustment and accommodation carry a price (allostatic load)
No pathology	Potentially leads to pathology

*^a^Anticipatory components of homeostatic control are usually restricted to small effects of, e.g., circadian rhythms, while allostasic anticipation results in profound adaptation in the awaiting of major strains of threats*.

Usually, two types of allostatic load are distinguished. *Type 1 allostatic load* occurs, if energy demands exceed the sum of energy intake and the amount of energy that can be mobilized from stores. Typical examples are breeding birds exposed to inclement weather conditions and the conflict of homeothermic animals resulting from reduced availability of food in cold seasons, when they have both to save energy and to increase energy spending on maintaining body temperature (101). *Type 2 allostatic load* results from *expected* increase in energy demand, although the cumulative energy balance is still sufficient. This constellation is typical of psychosocial stress situations, e.g., in predominating competitive social structures within animal populations and, as applied to humans, conflicts arising from differences in socioeconomic status (101). Multiple components of an unhealthy lifestyle including overnutrition, poor sleep, and toxic chemicals are also able to contribute to the phenotype of allostasis (107). Long-term consequences of type 2 allostatic load include obesity, hypertension, type 2 diabetes mellitus, endothelial cell damage, and dyslipidemia, i.e., classical components of metabolic syndrome.

Our group has proposed to extend the concept of allostasis to the adaptive response of thyroid function in defined straining situations (35). This extension goes beyond the simple issue of energy balance, although thyroid hormones have an intricate relationship to energy homeostasis. While stimulating the mobilization of energy for metabolic usage thyroid hormones also increase its consumption. Their activation is coupled with a depletion of reduced glutathione stores involved in regeneration of NADH and NADPH acting as cofactors of deiodinases (86, 87, 108–110). It may therefore be expected that when energy or glutathione availability does not meet their consumption, active thyroid hormones are selectively downregulated. By analogy to classical concepts we will subsequently refer to this situation as *type 1 thyroid allostasis*. Conversely, in situations, where energy stores have to be mobilized to meet *anticipated* demands (e.g., in pregnancy, endurance training and adaptation to cold weather conditions), upregulation of active thyroid hormones will be beneficial. Since these situations bear a resemblance to classical type 2 allostatic load, we will sum them up as *type 2 thyroid allostasis*. This is further justified because a high-T3 constellation in the absence of hyperthyroidism is also observed in obesity (111) and psychosocial stress (112), i.e., type 2 allostatic load according to the classic definition by McEwen and Wingfield.

In summary, unlike classical stress transduction systems the HPT axis produces two phenotypically distinct types of allostatic load if strained: in type 1 allostasis production of thyroid hormones, especially T3, is downregulated, while it is upregulated in type 2 allostasis. In that respect, the thyroid stress reaction differs sharply from that of the HPA axis, the prime example of an allostatically controlled system, as the latter responds invariantly with increased release of cortisol.

### Molecular Mechanisms of Thyroid Allostasis

In situations of starvation, inflammation, and oxidative stress, a variety of mediators (including nitric oxide, hydrogen peroxide, proteolysis-inducing factor, angiotensin II, TNF-α, and other cytokines) converge in the NF-κB pathway, a key regulatory system of immune response, cell proliferation, and apoptosis (113). Among multiple other effects the NF-κB/IL6 signaling pathway (114, 115) inhibits T3-induced expression of peripheral type 1 deiodinase. Downregulation of D1 and peripheral type 2 deiodinase (D2) results in reduced concentrations of free and total T3 (41, 116).

Recent research revealed a complex interaction of insulin and thyroid hormone signaling in skeletal muscle, which might also extend to lung and liver tissue (117, 118). *Via* the PI3K–mTORC2–Akt pathway insulin and IGF-1 inactivate FOXO1 by phosphorylation at Ser256, which leads to increased D2 activity (117). T3 again inhibits Akt activity, thereby closing a negative feedback loop (118). This mechanisms might play a pivotal role in linking reduced concentrations of insulin in fasting state (119) as well as decreased IGF-1 levels in a subgroup of critically ill (120) to hypodeiodination and consecutive low-T3 syndrome.

**Figure 5:**
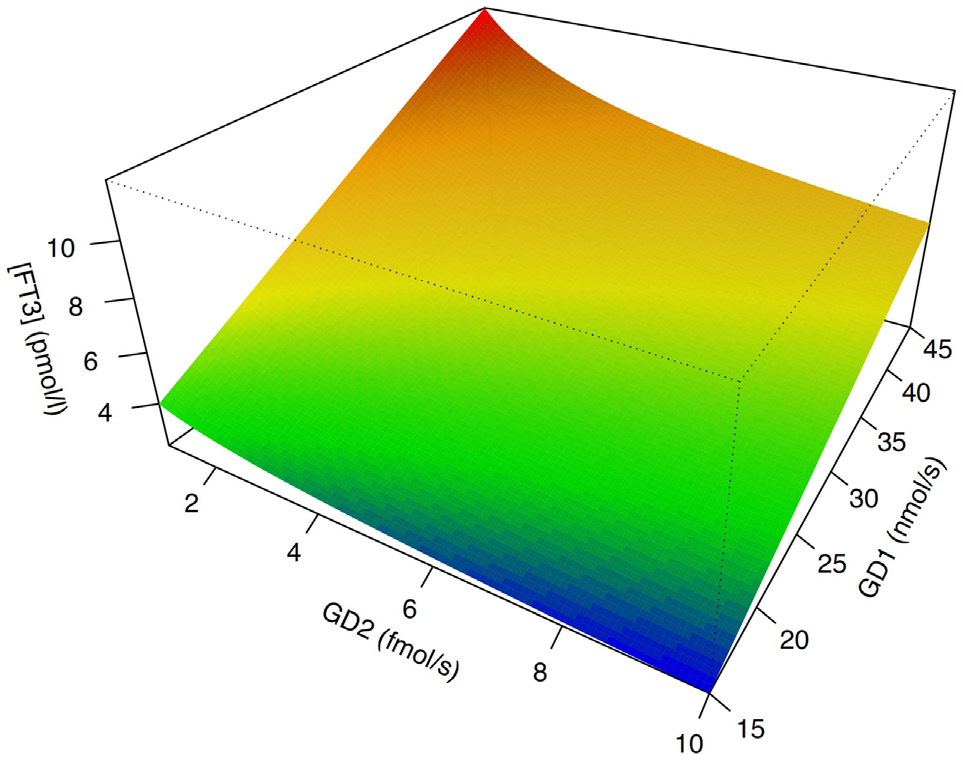
The phenotype of low-T3 syndrome may result from both peripheral hypodeiodination and central hyperdeiodination. Although FT3 concentrations rise with increasing sum activity of peripheral type 1 deiodinase (GD1), they descend with increasing activity of central type 2 deiodinase (GD2). This seeming paradox is explained by feedback effects (20, 21). Despite research in humans being hindered by ethical and methodological barriers, results of computer simulations [shown is sensitivity analysis based on SimThyr 4.0 (128)] and animal experiments (126, 127) are consistent.

In isolation, decreased peripheral step-up deiodination would lead to increased (disinhibited) TSH release, which would result in elevated serum thyrotropin concentration. This, in turn, would reset the concentrations of T3 to their previous levels, thus neutralizing the effect of hypodeiodination. However, concomitantly with reduced peripheral step-up deiodination, D1 and D2 located in hypothalamic tanycytes (95), and the anterior pituitary gland are upregulated. This change is mediated by bacterial lipopolysaccharide (121, 122), alterations in cytokines, e.g., IL-12 and IL-8 (43) and, possibly, increased concentrations of 3,5-diiodothyronine (61–63), triiodothyroacetate, and tetraiodothyroacetate (123, 124) during the acute phase response. The upregulation of central step-up deiodination results in increased central thyroid hormone signaling and, consequently, suppressed release of TRH and TSH. The seeming paradox that low-T3 syndrome may ensue from hyperdeiodination is resolved by the spatial diversity of deiodinase activity (Figure 5). This was investigated *in silico* by computer simulations (20) and confirmed by means of animal experiments *in vivo* (83, 84, 121, 122, 125–127).

A coordinated interaction of central and peripheral deiodinases in the lead-up to low-T3 syndrome is further supported by upregulation of type 3 deiodinase (D3) in TACITUS (129, 130), giving rise to elevated serum concentrations of rT3, a iodothyronine with inhibiting effects on thyroid hormone signaling. Increased D3 activity in starvation may be caused by decreased leptin concentrations as shown in mice experiments (23, 131). During prolonged critical illness, decreased food intake might be an important factor in regulating the activity of liver deiodinases (132). D3 in peripheral organs might also be upregulated by hypoxia due to decreased tissue perfusion during illness (133).

In addition, TSHb is decreased through IL-1b and TNF-α independently of T3 uptake and action in pituitary cells (134, 135). Moreover, supraphysiological concentrations of IL-1a and IL-1b suppress cAMP accumulation, thus inhibiting the TSH-induced Tg mRNA expression and Tg release in human cultured thyrocytes (136, 137).

Thyrotropin-releasing hormone neurons originating from the hypothalamic PVN, the major autonomic output area of the hypothalamus (95, 138–140), play a key role in the central control of thyroid homeostasis by providing a set point for the short loop control (141). The release of TRH is inhibited by central T3, which is predominantly generated by D2 expressed in tanycytes lining the third ventricle (142–145) (Figure 3). This circuit forms an additional long feedback mechanism of thyroid homeostasis (83, 84). Apart from the so defined cascade control mechanism, TRH neurons contribute to the coordination of global energy metabolism by integrating multiple afferent signals (146) including catecholamines (147, 148), cocaine- and amphetamine-regulated transcript (149, 150), leptin (151), and alpha-MSH (146, 152) (all stimulating) as well as neuropeptide Y (NPY) (153–155), agouti-related peptide (141, 146, 156), and glucocorticoids (146) (all inhibiting). Moreover, endocannabinoids have been shown to exert inhibitory effects on TRH neurons *via* the type 1 cannabinoid receptor (97). Animal experiments revealed that the integration of all afferent projections has profound effects on secretion of TRH and consecutively TSH in straining situations (95).

Depending on the origin of stress (physical or psychogenic), its duration and the animal’s endocrine and energetic status TRH release may be upregulated or downregulated (157) subsequently affecting the set point of the overall homeostatic system. Downregulation with consecutive thyrotropic adaptation, i.e., low or normal TSH levels despite low concentrations of T4 and/or T3, may occur for instance in cases of systemic infection and sepsis, where lipopolysaccharides induce D2 activity in tanycytes (126). Moreover, low TRH expression in the PVN characterizes the NTIS (95). In addition to the effects of NPY as neurotransmitter, elevated fasting serum concentrations of NPY have a stimulatory effect on hepatic thyroid hormone degradation *via* increased glucuronoconjugation (facilitating biliary clearance) and sulfoconjugation (enabling step-down deiodination to rT3S) (140, 158).

A high proportion of the circulating iodothyronines is bound to thyroxine-binding globulin (TBG), transthyretine, and albumin. This mechanism contributes to the exceptionally long half-lives of thyroid hormones. In case of rapid onset of stress situations, e.g., severe illness, the time frame of the described control mechanisms would be too long to be effective, if plasma protein binding of thyroid hormones remained unchanged. However, in critical illness the extent of plasma protein binding is reduced owing to decreased concentrations of binding proteins and the existence of certain binding inhibitors (57, 58, 159). This effect is putatively mediated *via* cytokines (160). Consequently, degradation of iodothyronines is considerably accelerated, which represents another underlying mechanism toward the adaptation of the feedback loop to conditions of type 1 allostatic load (161–163).

In an animal model for prolonged critical illness the iodothyronine membrane transporters MCT10 and OATP1C1 (but not MCT8) were increased, suggesting some adaptation at the level of transmembrane transport, however, with uncertain clinical relevance (164).

Finally, alternative metabolic pathways of thyroid hormones in peripheral tissues such as sulfation, conjugation to bile acids and glucuronide, and ether link cleavage may affect the concentrations of thyroid hormones in critical illness (165–169).

## Thyroid Allostasis in Various Physiological and Pathological Conditions

A variety of scenarios associated with type 1 and type 2 allostatic load have been recognized to result in adaptive changes in thyroid homeostasis. Table 2 provides a short summary, and the associated conditions are subsequently described in more detail.

**Table 2:** Characteristic phenotypical changes of thyroid-stimulating hormone (TSH) and various classical and non-classical thyroid hormones in certain allostatic situations show nearly opposing changes in type 1 and type 2 allostatic load (61, 63, 111, 123, 170–182).

	TSH	FT4	TT4	FT3	TT3	rT3
**Type 1 allostasis in fetal period, acute and chronic critical illness, and in deprivation of energy**						
Fetal life	↓, → or ↑	↓	↓	↓	↓	↑
Caloric deprivation	→ or ↓	→	↓	↓	↓	↑
Exhausting exercise	→ or ↓	→	↓	↓	↓	↑
Critical illness (general)	→ or ↓	→	→ or ↓	↓	↓	→ or ↑
Chronic heart failure	→ or ↓	→	→ or ↓	↓	↓	→ or ↑
Renal diseases	→	→ or ↓	→ or ↓	→	↓	→
Liver diseases	→	→ or ↓	↑	↓	→ or ↑	→ or ↑
Pulmonary diseases	→	→	→	↓	→	→ or ↑
Diabetes mellitus	→ or ↓	→ or ↑	↓	↓	↓	→ or ↑
Sepsis	↓	→	→ or ↓	↓	↓	→ or ↑
HIV infection	→	→ or ↓	→	→ or ↓	→	→ or ↓
Depression	→	→ or ↑	↑	↓	↓	→ or ↑

**Type 2 allostasis-related conditions**						

Pregnancy	→ or ↓	→	↑	→	↑	→
Endurance training	↓, → or ↑	↑	↑	↑	→ or ↓	↑
Obesity	↑	→ or ↓	→ or ↑	↑	↑	→ or ↓
Adaptation to cold	↓, → or ↑	↑	↓, → or ↑	↑	↑	→ or ↓
Acute schizophrenia	→ or ↑	→ or ↑	↑	→ or ↑	↑	→
Post-traumatic stress disorder	→	→	↑	↑	↑	?

*FT4 and FT3, free T4 and T3, respectively; TT4 and TT3, total (free + protein-bound) T4 and T3, respectively; rT3, reverse T3; TSH, thyroid-stimulating hormone*.

*Hormone concentration unchanged (→), increased (↑), decreased (↓), or not reported (?). Small studies also reported increased concentrations of 3,5-diiodothyronine (3,5-T2) (61–63), triiodothyroacetate and tetraiodothyroacetate (123, 124), and decreased concentrations of 3-monothyronamine (63) in critical illness and chronic heart failure (not shown in table). See text for definition of type 1 and type 2 allostasis in the context of pituitary–thyroid function*.

### Energy Restriction and Starvation

**Figure 6:**
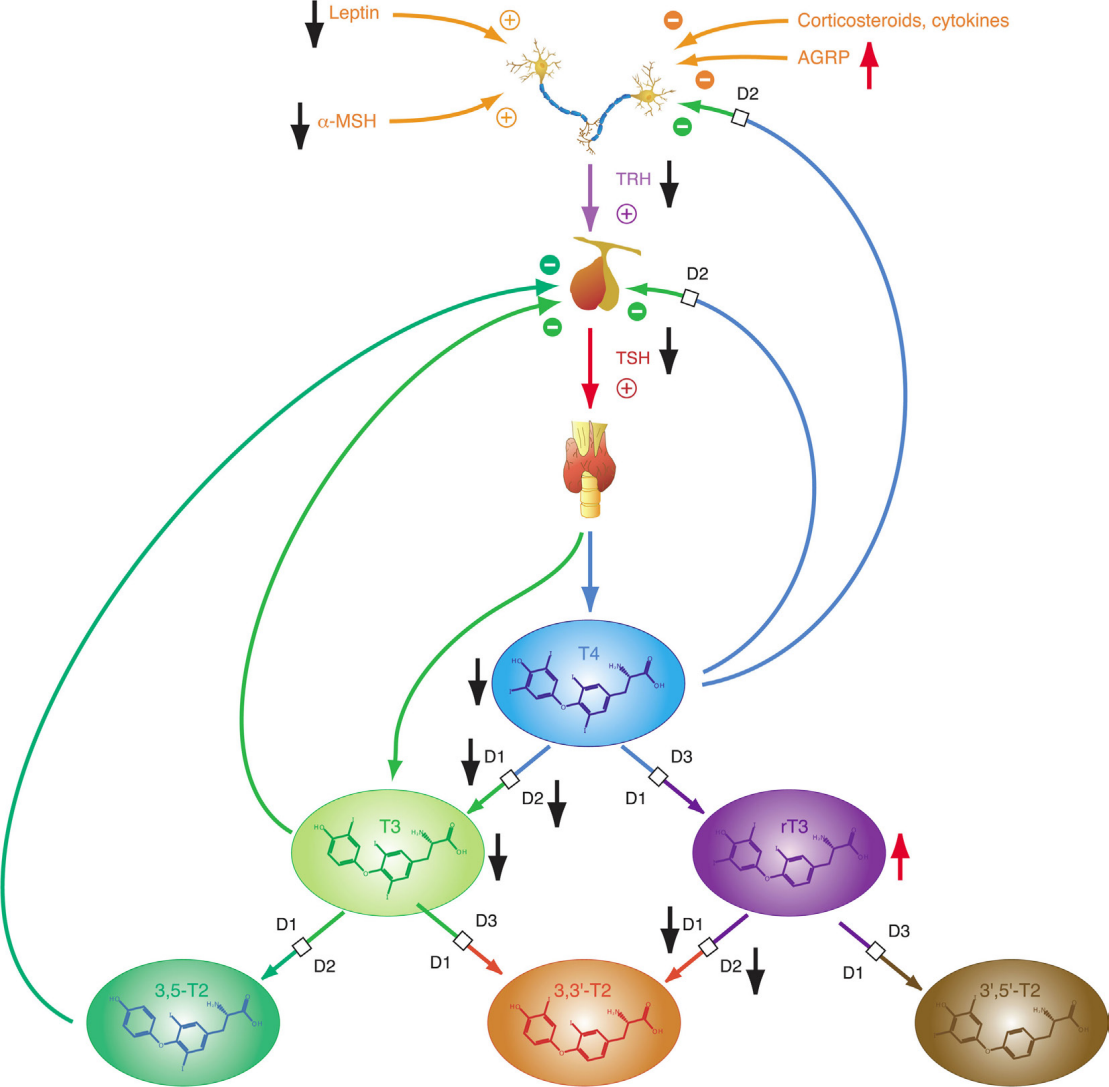
In starvation, both step-up deiodination (*via* D1 and D2) and thyroid-stimulating hormone (TSH) release are reduced, leading to low-T4 and low-T3 constellations. rT3 concentrations may be increased. Black and red arrows indicate the direction of change from normal, homeostatic conditions in fed state.

Early reports that serum concentrations of T3 are reduced in states of low caloric intake (47, 49) gave rise to the concept of what we now call the Low-T3 syndrome. Reduced T3 concentrations have been described in various conditions associated with energy deprivation, including anorexia nervosa (183–185), calorie-free diet in obesity (47), military combat training with caloric restrictions (186, 187) and other energy-deficient situations (188). Even moderate weight loss may result in hypodeiodination with consecutive decreased T3 concentrations (189). Today, at least three mechanisms explaining this finding are known (Figure 6) (23). In the fed state, peripheral step-up deiodination is stimulated by insulin (119) and bile acids (190–192). In addition, increased leptin concentrations facilitate release of TRH and TSH *via* the hypothalamic melanocortin pathway (23, 83, 146, 193). Together, these different mechanisms enhance conversion of T4 to T3, thus mediating postprandial thermogenesis. Conversely, in fasting conditions concentrations of insulin, bile acids, and leptin are low, which results in decreased step-up deiodination and thyrotropic adaptation, and eventually in low-T3 syndrome. Additional mechanisms leading to impaired TSH release include increased expression of neuromedin B, a bombesin-related peptide, which is an inhibitor of TSH secretion, and upregulation of hypothalamic D2 expression during fasting (23), resulting in low TRH expression in the PVN (25, 95, 98, 194).

### Obesity

**Figure 7:**
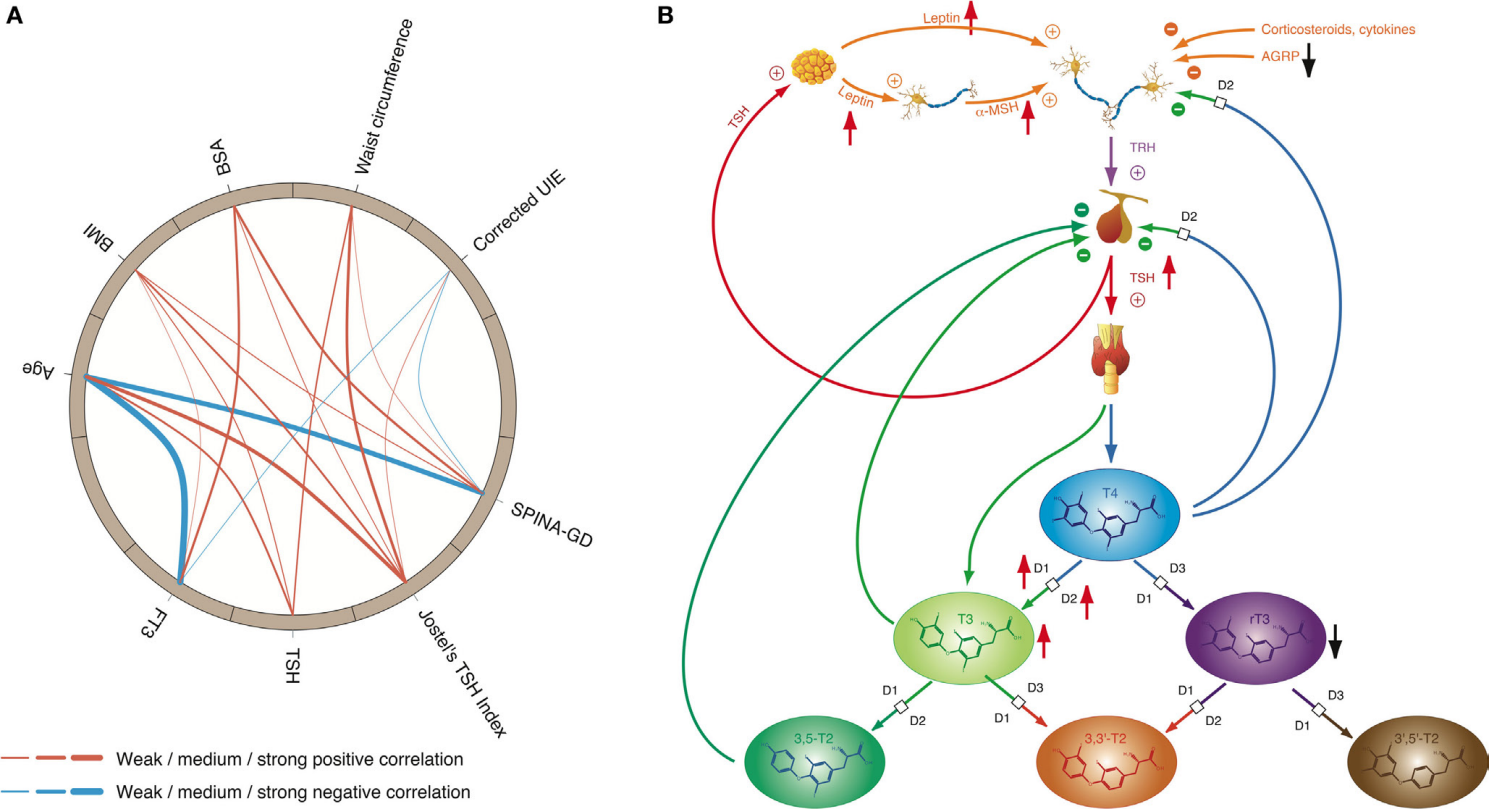
High-T3 concentrations (although in most cases within the reference interval), reduced rT3 concentrations, increased activity of type 1 and type 2 deiodinase, and comparatively high thyroid-stimulating hormone (TSH) levels are the typical signature of obesity, a classical phenotypical sequela of type 2 allostatic load. **(A)** In healthy participants of the NHANES program (200), concentrations of TSH and FT3 as well as Jostel’s TSH index (a measure for the set point of thyroid homeostasis) and SPINA-GD (an estimate for total deiodinase activity) show a significantly positive correlation to body mass index (BMI) and waist circumference, and with the exception of TSH also to body surface area (BSA). In addition, FT3 and SPINA-GD correlate negatively to age, and minor associations exist to creatinine-corrected urinary iodine excretion (UIE). In this circular map, positive correlations are marked in red and negative correlations in blue. The widths of the splines represent the correlation coefficient to denote the strengths of association (201). **(B)** Mechanisms of adaptive responses in obesity are mediated by elevated leptin concentrations and increased alpha-MSH signaling, while activity of AGRP terminals is reduced.

Obesity is a classical consequence of type 2 allostatic load (101). It is linked to multiple metabolic and endocrine responses (111) including thyroid function. The interconnection between thyroid hormones and body weight is bidirectional, and both hypothyroidism and hyperthyroidism are known to result in changes of body mass. Conversely, obesity may result in adaptive responses of thyroid homeostasis: a variety of studies, as recently reviewed by Pacifico et al. (195) and Fontenelle et al. (111), described elevated TSH levels and increased total step-up deiodinase activity (although predominantly within the reference range) in patients with weight gain, while concentration of rT3 has been reported to be decreased. Reversibility of these alterations after weight loss indicates that they are consequence rather than cause of overweight (196). A recent study (42) described a significant rise in TSH in the absence of peripheral hypothyroidism in men with non-metastatic prostate cancer undergoing androgen deprivation therapy. The effect was mediated by body composition changes and by the fat-associated hormone leptin rather than androgen deficiency. Another study interrelated non-alcoholic fatty liver disease with higher fT3 concentrations in euthyroid subjects, probably consequent to central obesity (197). Both central and peripheral components of the feedback loop are apparently involved in the reactive adjustments to obesity (Figure 7). Increased concentrations of adipokines such as leptin have been proposed to be a key element of obesity-related thyroid allostasis, but mitochondrial dysfunction (195, 198), chronic inflammation, and insulin resistance (199) as well as both central and peripheral resistance to thyroid hormone may play additional roles (111).

Since thyroid hormones are potent stimulators of adaptive thermogenesis (202–204), upregulation of TSH release and deiodinase activity stimulates dissipation of energy and therefore may be part of autoregulatory mechanisms of body mass and fat storage. On the other hand, some of the obesity-related changes in thyroid function may contribute to the unfavorable metabolic phenotype of overweight (111). This and significant cardiovascular side effects of hyperthyroidism are the main reasons that intake of thyroid hormones is strongly discouraged as an adjunct in the treatment of obesity (205).

### Adaptive Thyroid Responses to Thermoregulatory Challenge

In mammals, thyroid hormones are potent mediators of efficient thermoregulation. This ensues from a complex mechanism tightly integrating deiodinase activity with sympathetic signals (206), which leads to upregulation of the protein UCP1 in mitochondria of skeletal muscle and brown adipose tissue (207–209). This results in uncoupled oxidative phosphorylation and finally non-shivering thermogenesis (202–204, 210, 211). It is therefore not surprising that mammals, typical examples for homeotherm animals (endotherm thermoregulators), usually exhibit elevated serum concentrations of T4 and T3 in winter and during hypothermia as long as they are sufficiently fed (212, 213). Increased concentrations of TSH and/or thyroid hormones in cold seasons and during hypothermia have also been described in humans (214–217). Although the mechanism of thyroid hormone-mediated expression of UCP1 plays a pivotal role in efficient thermoregulation of mammals (202, 218, 219) it has not been described in non-mammal homeotherm vertebrates, e.g., in birds, and it is probably inexistent in homeotherm arthropods. Of note, although some insect species, e.g., *bombus* and *apis*, are homeotherm (220, 221), their temperature regulation is less potent and less energy efficient than that of mammals. This may be in part due to the fact that they probably lack endogenous production of thyroid hormones (222–224).

In hibernating mammals, the situation is different to that of non-hibernating mammals, since here concentrations of T4 and T3 are downregulated during hibernation (78, 225–228). This also applies to the endocrine response to cold in starving mammals (229) and poikilotherm vertebrates during torpor (79).

In summary, during cold periods T3 and T4 are upregulated in fed non-hibernating homeotherm mammals, but in an NTIS-like pattern downregulated in hibernating mammals, starving non-hibernating mammals and poikilotherm vertebrates. Both mechanisms support conservation of energy: the first one by making thermoregulation more efficient, and the second one by partly tuning down the metabolism in periods of lower demand and supply.

### Fetal Life

**Figure 8:**
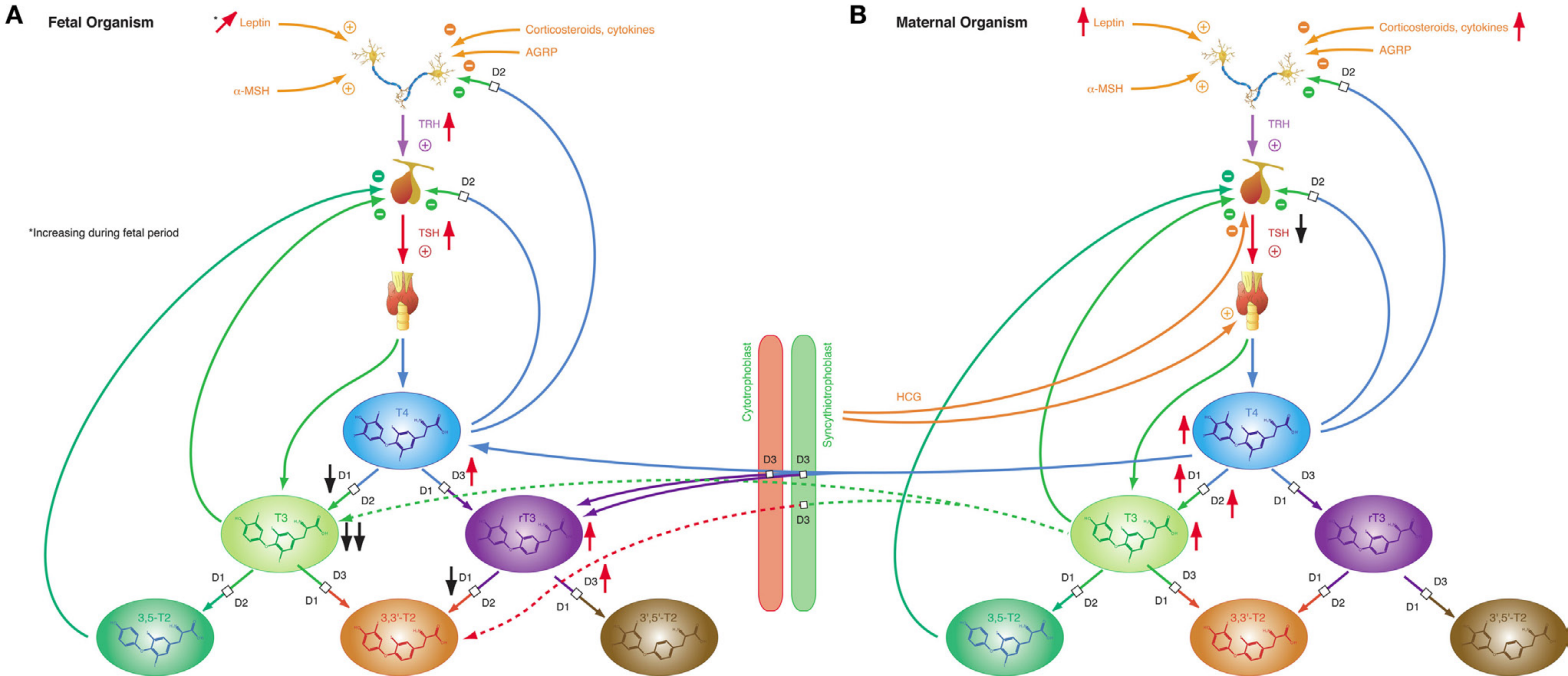
Fetal and maternal thyroid homeostasis are dovetailed to optimize conditions for both organisms. **(A)** After maturation of the feedback loop in the 20th week of gestation, fetal thyroid-stimulating hormone (TSH) concentrations and step-down deiodination *via* D3 are temporarily increased, while step-up deiodination is decreased. This results in a pattern of markedly reduced T3 concentrations and elevated rT3 levels. Black and red arrows indicate the difference compared to normal, homeostatic conditions in healthy newborns and adults. **(B)** Pregnancy is accompanied by a characteristic “anti-NTIS”-like constellation of thyroid homeostasis including high concentrations of T3 and T4, step-up hyperdeiodination and increased binding of thyroid hormones to plasma proteins.

Iodothyronines are critical for development in the embryonal and fetal periods. Both hypothyroidism (230) and oversupply with thyroid hormones (231) may result in fetal loss and severe developmental disorders. In humans, the fetal thyroid gland starts to secrete hormones in the beginning of the 12th week of gestation (29). However, the feedback loop begins to be functional in the 20th week, i.e., in mid-gestation (29). In the first half of pregnancy, the fetus is largely dependent on maternal supply with thyroid hormones, probably explaining, why production of iodothyronines is upregulated in the mother (230). Despite this anti-NTIS-like pattern in the maternal metabolism, which is mainly mediated *via* human chorionic gonadotropin (hCG) and estrogens (230), concentrations of free and total T3 are low in the fetus throughout gestation, and concentrations of TBG, free and total T4 are, although rising with increasing gestational age (230), lower in the fetal than in the maternal circulation (Figure 8) (232). TSH levels attain a maximum of about 15 mIU/L in the 20th week, when the feedback loop maturates, and then again at birth (29). Both in fetal serum and in amniotic fluid, concentrations of rT3 are markedly increased (230), which probably results from high activity of type 3 deiodinase in placental tissue and multiple fetal organs (230). Increased concentrations of sulfated metabolites of iodothyronines result from low type 1 deiodinase activity in fetal tissues and because T4 sulfate and T3 sulfate are not substrates for placental type 3 deiodinase (230). At birth, concentrations of TSH, T4, and T3 sharply spike to attain slightly elevated levels in the neonatal metabolism (29).

These patterns of thyroid hormones in the normal fetus closely resemble the constellation of NTIS. The conclusion that the normal fetal concentrations of thyroid hormones are beneficial despite being markedly different from that of both healthy adults and healthy newborns is supported by the observation that this constellation is actively defended in situations of thyroid disorders (233): in hypothyroid fetuses D2 activity increases and activities of D1 and D3 decrease, thus providing support for shunting of T4 to brain tissue (230), while, on the contrary, elevated concentrations of iodothyronines stimulate the activity of D3, which results in increased degradation of active thyroid hormones to rT3, 3,3′-T2 and 3′,5′-T2 (234–236).

### Pregnancy

The adaptive endocrine response in pregnancy manages a trade-off situation, where the maternal organism is faced with the dual challenge of optimizing conditions for the developing fetus and its own survival. Allostatic changes are mediated both by the central hypothalamic–pituitary unit and the fetal-placental unit. The latter secretes protein and steroid hormones that modify the function of endocrine organs in the mother’s organism throughout pregnancy (237). Due to its high structural similarity with TSH, the glycoprotein hormone hCG stimulates the human TSH receptor, which enables the placenta to gain parallel control over the thyroid system in early gestation (238). In extreme situations, e.g., starvation, the adaptive gestational responses control type 1 allostasis. Mostly, however, resources are sufficient to permit an anticipatory endocrine response of type 2 allostasis. Typical responses of the pituitary–thyroid axis in pregnancy include enhanced secretion of thyroxine from the thyroid gland and increased step-up deiodination. Unlike in obesity or other states of type 2 allostasis, TSH concentrations are low-normal or slightly decreased (230) (Figure 8B). This is a consequence of both elevated concentrations of T4 and hCG (stimulating TSH receptors in the anterior pituitary gland). In addition, plasma protein binding of thyroid hormones is increased. The majority of these effects are mediated by hCG, which displays, in addition to its gonadotropic action, mild TSH-mimicking effects in its sialylated form and TSH-antagonistic effects in a desialylated variant (239). Thyroid overstimulation by hCG is possible in pregnant women in the first trimester resulting in a distinct entity of gestational hyperthyroidism or hyperthyroidism in trophoblastic diseases, which may also affect men with testicular cancer (238, 240–247). This scenario exceeds physiological adaptation and translates into a specific disease entity. It demonstrates the strength of hCG-mediated effects, which confer a not so rare risk of subclinical hyperthyroidism in otherwise normal pregnancies (238).

The “anti-NTIS”-like pattern of maternal thyroid homeostasis in pregnancy results in increased availability of thyroid hormones for the developing fetus, which is especially necessary in the early phases of gestation, when the fetal thyroid is still unable to produce sufficient amounts of thyroid hormones, the more as the transport capacity through the placental barrier is limited.

Pregnancy-related allostatic changes in thyroid function are frequently causing problems in the differential diagnosis of thyroid disease as described below in the Section “Methods of Assessment and Differential Diagnosis.” Because a sufficient supply with levothyroxine is critical for the development of the fetus, thresholds for initiation of substitution in subclinical hypothyroidism and dosage adjustment in hypothyroid women taking L-T4 prior to pregnancy had been lowered by professional societies (248–251). Based on evidence the American Thyroid Association and the Endocrine Society had recommended to lower the upper range of the TSH reference range to 2.5, 3.0, and 3.5 mIU/L in the first, second, and third trimester of pregnancy, respectively. Although several observational studies reported adverse pregnancy outcome in even mild hypothyroidism (252–261), two recent interventional trials did not confirm a beneficial effect of substitution therapy in subclinical hypothyroidism and hypothyroxinemia with respect to children’s IQ at age of 5 years and secondary outcome markers (262, 263). Consequently, the recommended upper limit of the TSH reference range has been raised back to 4 mIU/L in the most recent guideline issued by the American Thyroid Association (264). However, thyroid peroxidase antibodies should be measured in pregnant women with TSH concentration above 2.5 mIU/L and treatment be considered, if antibody titers are positive, even if TSH levels are between 2.5 and 4 mIU/L (264).

The reason for these discrepancies and the lack of success by interventional studies is unknown. Ethnic differences among study populations (265) and the inverted U shape of the relation between maternal FT4 concentration and child IQ (266–268) as well as the relatively late onset of substitution therapy after the 10th week of gestation in the reported substitution trials (262, 263) may play a role. This uncertainty warrants further research.

### Exercise

Intensive muscle activity in sports and training is associated with profound changes in endocrine control (27) and cytokine patterns (269, 270). This suggests modifications of thyroid homeostasis during or after exercise.

**Figure 9:**
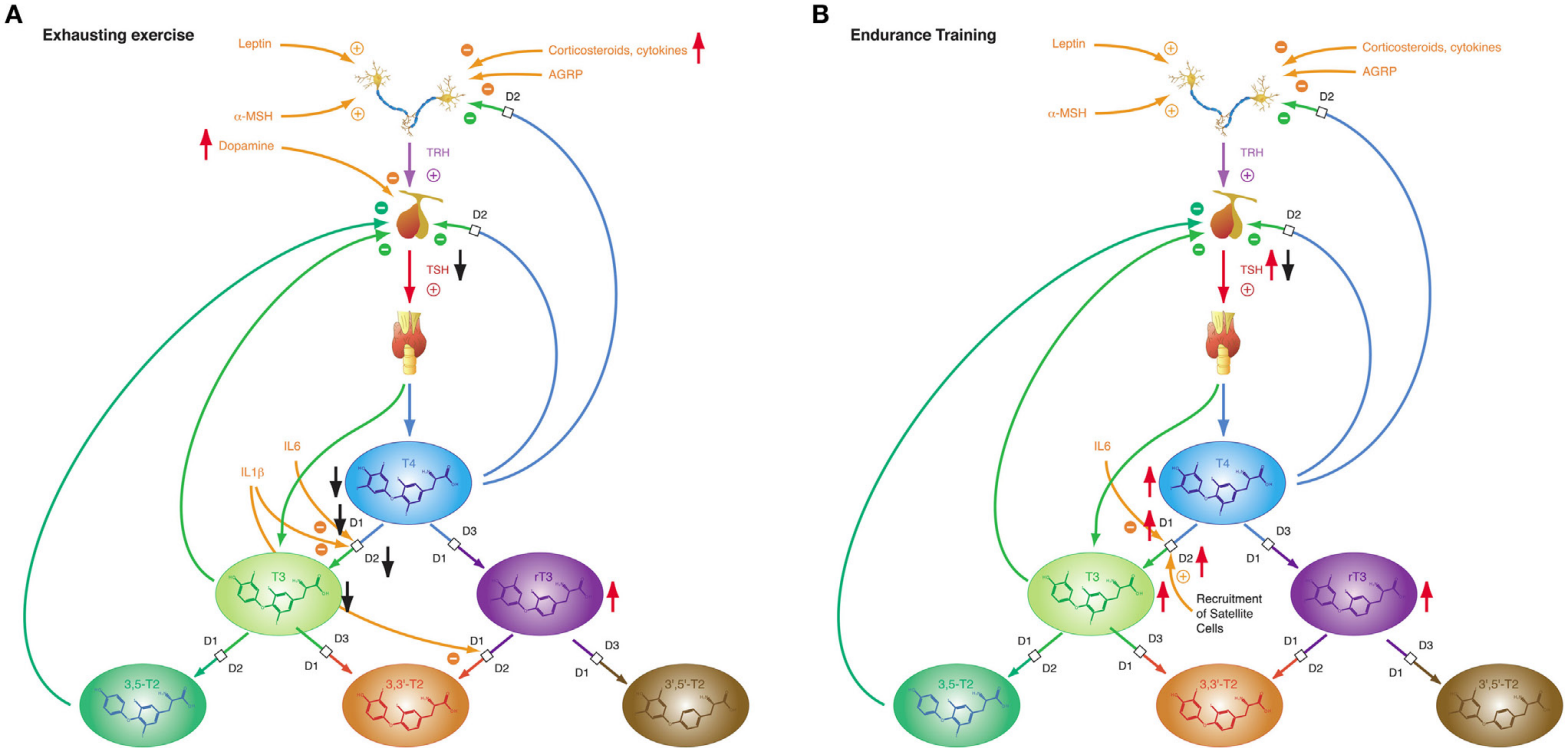
The adaptive response of the hypothalamic–pituitary thyroid axis to exercise is heterogeneous, depending on duration and intensity of training and on the interval between exercise and laboratory investigations (27). This diversity may result from pre-analytical factors (e.g., hemoconcentration) and from an overlap of type 1 and type 2 allostatic load. **(A)** Exhausting exercise. **(B)** Endurance training.

The response of thyroid hormones to exercise varies (Figure 9). With a few exceptions (271–273), most studies investigating thyroid hormones during or in a short-time interval after training found elevated concentrations of TSH, T4 and/or T3 (46, 274–276). After resting or in prolonged training programs with repeated heavy strain, however, the majority of studies described reduced concentrations of TSH, T4, and T3 (186, 187, 277–281). This seeming contradiction was attributed to hemoconcentration in or after exercise leading to falsely elevated hormone concentrations in short-time exercise (27). This assumption is supported by trials, where thyroid hormones have been measured both on short and long timescales. Hormone concentrations, while elevated during physical strain or at exhaustion decreased at rest after exercise (46, 186, 275–277). Resting allows for rehydration and represents a more realistic situation. As a consequence it has been recommended to allow for a 24-h recovery period before participants report for laboratory testing (282).

Of note, TSH and thyroid hormone concentrations are elevated in endurance exercise and in the beginning of military combat training before exhaustion (186, 277, 283), even if allowance was made for resting and rehydration before investigation (283). With the onset of exhaustion, the pattern changes and hormone concentrations decrease to subnormal values (186, 187, 277). This suggests that the variable homeostatic response to exercise may possibly result from a second mechanism, where type 1 allostasis ensues from exhaustion and deprivation of energy, thereby leading to downregulation of TSH and peripheral thyroid hormones, whereas in endurance training and before exhaustion allostasis is shifted to type 2 and stimulated release of TSH, T4 and T3.

The NTIS-like pattern of thyroid hormones after exhausting training is confirmed by two studies showing increased rT3 concentrations (187, 283) in physical strain. The type 1 allostatic endocrine responses were more pronounced in military combat training programs, when participants were subject to additional deprivation of sleep and energy (186, 187, 277).

### Acute and Chronic Critical Illness

Characteristic patterns of NTIS have been described in a multitude of acute and chronic somatic illnesses including states of shock (284), circulatory arrest (285, 286), respiratory failure (65), community-acquired pneumonia (287), sepsis (288), chronic respiratory (289, 290) and cardiovascular (61, 64, 66, 291, 292) disease, renal failure (293–298), COPD (289), gastrointestinal diseases (299–301), autoimmune diseases (71, 302, 303), and cancer (130, 290, 304). Phenotypes of NTIS with high and high-normal FT4 concentrations have been described in dementia and frailty in elderly persons (305).

**Figure 10:**
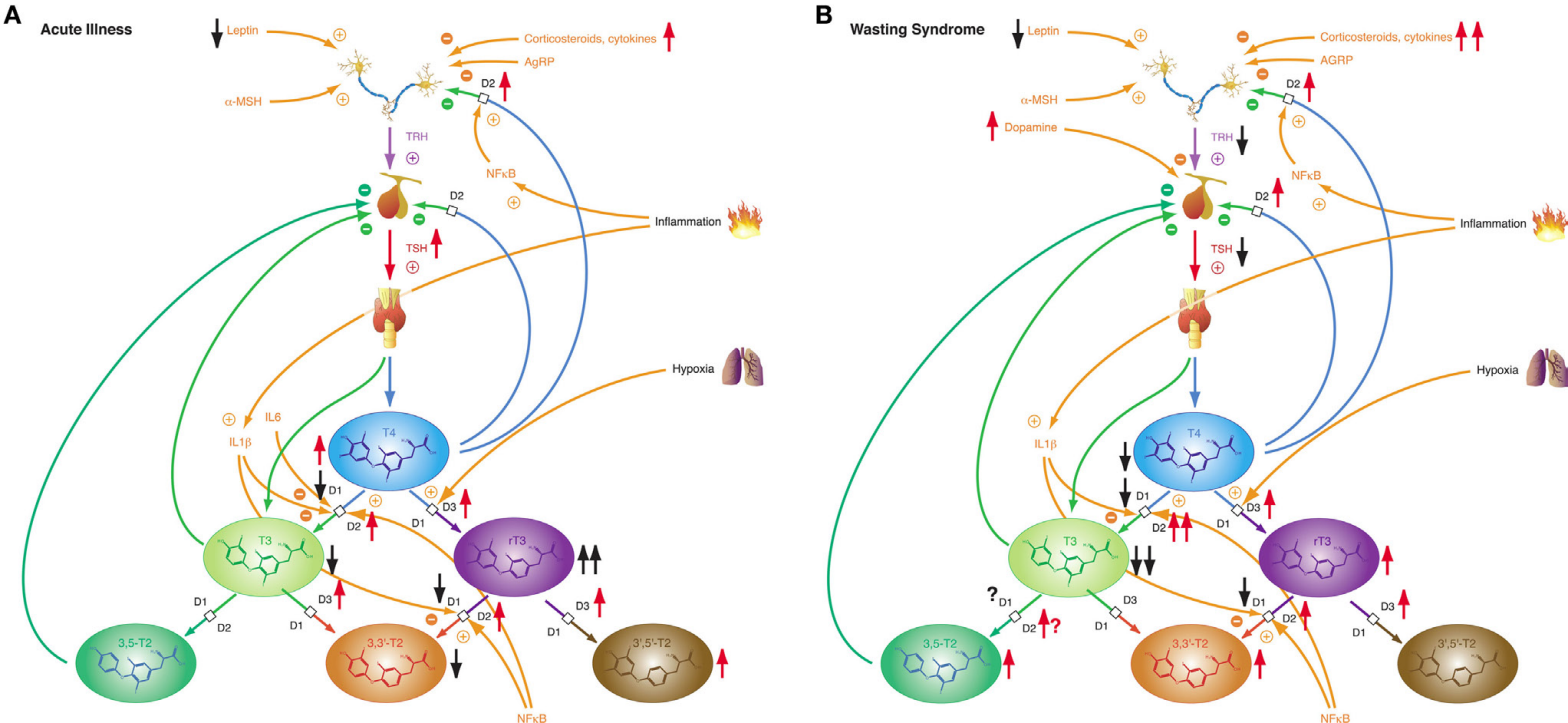
Depending on severity and duration of disease, non-thyroidal illness syndrome presents with two related, but distinct phenotypes. **(A)** Allostatic reactions of the pituitary–thyroid feedback control system in acute illness lead to slightly decreased T3 and 3,3′-T2 concentrations, slightly elevated T4 and 3′,5′-T2 levels and markedly increased rT3 concentrations. **(B)** Medium-term and long-term adaptations in ongoing illness (“wasting syndrome”) and chronic disease result in thyrotropic adaptation and slight increases of rT3, 3,3′-T2, and 3,5-T2 concentrations concomitant to low-T3 syndrome.

While most forms of acute critical illness may be interpreted as a state of starvation, the chronic form of severe illness—a result of modern critical care—represents most commonly a state of adequate nutrition (306). Hence it is not surprising that acute and chronic critical illness elicit different phases of NTIS: the acute phase (Figure 10A) and the chronic or prolonged phase (Figure 10B), also referred to as *wasting syndrome* (307). The former seems to beneficially affect outcome, the latter to have an impairing effect (308).

Non-thyroidal illness syndrome is a disease-independent risk factor for survival, so it is important to understand the underlying mechanisms (60). Patients with low free T3 show a significantly higher mortality and a significantly longer duration of mandatory ventilation (56, 309). Furthermore, low free T3 is a strong prognostic predictor in B-cell lymphomas (310). The alterations of the acute phase of NTIS in critical illness occur within hours or days and are defined by increased release of anterior pituitary hormones, low levels of anabolic peripheral effector hormones, reduced thyroid hormone-binding protein concentration, reduced binding affinity, reduced expression of thyroid hormone transporters, decreased thyroid hormone uptake and altered expression of D1 and D3 activity and the thyroid hormone receptor alpha1 (TRα1). The prolonged phase, on the other hand, is characterized by a suppression of the hypothalamic-anterior-pituitary-peripheral-hormone axis and low levels of anabolic peripheral effector hormones. Peripheral tissues respond by reactively increasing the expression of monocarboxylate transporters, upregulating D2 activity, reducing D1 activity, and increasing sensitivity to thyroid hormone receptors. The chronic phase is characterized by a loss of pulsatility of TSH secretion, a reduced TRH-gene expression in the hypothalamic PVN, and suppressed hypothalamic stimulation. However, the pathogenesis is unclear. Altered D3 activity and MAPK and hedgehog pathway seem to play a pivotal role in the whole process (311). Despite our current knowledge, critical questions remain unanswered.

### Psychosocial Stress and Psychiatric Diseases

The relationship between thyroid hormones and psychological phenomena is a paramount example of mutual and reciprocal influences of mind and body. It had been recognized for more than 150 years that diseases of the thyroid are frequently accompanied by psychiatric symptoms (312, 313). Although depression is a classical symptom of hypothyroidism, and psychosis may result from thyrotoxicosis, the linkage is clearly bidirectional (314). A great number of studies over the last decades showed that characteristic changes of thyroid hormone concentration may arise from mental or psychological disorders (28, 314) in the absence of thyroid disease. This assumption is confirmed by interventional studies that show normalization of formerly changed hormonal parameters after the underlying psychiatric disease has been successfully treated.

As early as in 1968 John Mason predicted thyroid hormone concentrations to rise in response to psychosocial stress (315). Subsequent research revealed that the interaction is complex and non-linear and that it is additionally dependent on the nature of the underlying psychiatric disease. A recent study demonstrated that stress could trigger the onset and the recurrence of hyperthyroidism in patients with Graves’ disease (316). However, hyperthyroxinemia is indeed a nearly universal observation in different expressions of psychiatric disorders (317–322).

A large body of studies reported a characteristic pattern of thyroid hormones in MD (314, 323). Concentrations of T4 or FT4 are commonly increased during depression (324–329) and revert after recovery from MD, irrespective of the modality of treatment (330–339). Despite the presence of elevated T4 concentrations TSH levels tend to be normal in depressive patients, but circadian variation of TSH concentration is impaired (339–342) and the response to TRH test is blunted (323). Both total and free T3 concentrations are reduced in MD (175, 176, 343), but elevated in bipolar I disorder (237, 344). Concomitantly, rT3 concentrations are temporarily increased in both MD and manic disorder; however, not in bipolar I disorder (334, 345, 346).

Similar to the situation in bipolar I disorder, concentrations of free and total T3 are frequently elevated in post-traumatic stress disorder (PTSD) (177–179, 181, 182, 347), another classical example of a type 2 allostatic reaction (112). Except for concomitant borderline personality disorder (348), the common high-T3 syndrome in PTSD is at least partly due to increased step-up deiodination (179, 181, 182). Two studies in combat veterans of World War II and the Vietnam war revealed levels of total T4 and both total and free T3 to significantly correlate to severity of PTSD (181, 349). This anti-NTIS-like pattern is complemented by elevated TBG concentrations and increased plasma protein binding in these patients compared to healthy controls (179). Despite an elevated set point of thyroid homeostasis the response to TRH stimulation is blunted in PTSD (350).

A small number of studies reported elevated concentrations of FT4, TT3, and FT3 in schizophrenia spectrum disorders. This observation was, however, not reproducible in all studies and apparently dependent on severity of symptoms and the time after admission (351, 352).

In summary, MD is accompanied by a partly NTIS-like pattern, whereas bipolar I disorder and PTSD as well as severe and newly diagnosed schizophrenia involve a hormone constellation typical of type 2 allostasis. A relatively high set point for T4 is shared by all four disorders, as evidenced by unsuppressed TSH levels despite high T4 concentrations, whereas the response to the TRH test is mitigated.

**Figure 11:**
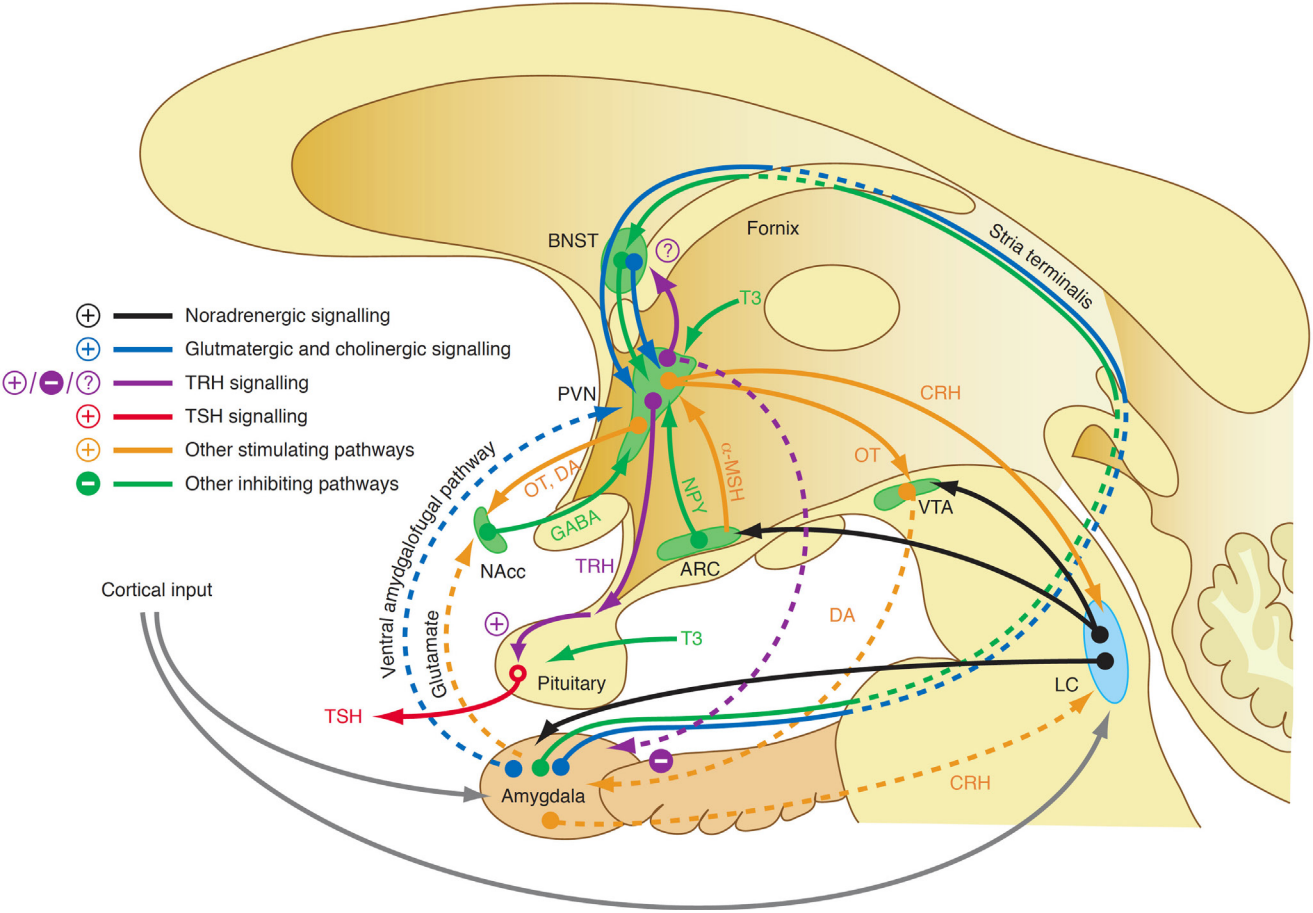
A complex interaction of positive and negative feedback mechanisms linking centers of the limbic system to hypothalamic nuclei explains the adaptive response of the hypothalamic–pituitary thyroid loop in type 2 allostasis resulting from psychosocial stress situations (355, 356, 358, 359). ARC, arcuate nucleus of the hypothalamus; BNST, bed nucleus of the stria terminalis; CRH, corticotrophin-releasing hormone; DA, dopamine; GABA, gamma-aminobutyric acid; LC, locus coeruleus; NAcc, nucleus accumbens; OT, oxytocin; VTA, ventral tegmental area.

Although our knowledge of the precise mechanisms mediating the endocrine response in this class of affective disorders is still limited, recent research revealed some elements that may play a key role in this scenario. For a long time, it was assumed that central TRH, which has in addition to its endocrine function widespread neurotransmitter and neuromodulatory effects, has a pivotal function in the link between depression and altered thyroid hormones. While TRH concentrations in CSF are increased in depressed patients (353, 354) and TRH levels are particularly high in subjects with violent suicidal behavior (353), the results after treatment and in recovery are inconsistent (323). A decisive influence possibly lies in the spatial distribution of neuromodulators and the complex interaction of positive and negative feedback loops between the limbic system and hypothalamus (Figure 11). TRH expression is upregulated in the amygdala in response to stress (355) and amygdala kindling (356), but downregulated in hippocampus (357). *Via* two pathways, such as the stria terminalis and the ventral amygdalofugal pathway, the amygdala stimulates the PVN, the origin of hypophysiotropic TRH neurons, with cholinergic and glutamatergic terminals (358). As a consequence, TSH release increases in situations of stress-induced type 2 allostasis (359). Of note, amygdala activity is inhibited again by means of feedback loops mediating the anxiolytic-like effect of TRH (360). In addition, the activity of the feedforward path is sensitive to context and circadian conditions, too (361). This complexity warrants further research.

From a teleological perspective, the type 1 allostatic pattern in depression makes sense. Depression and starvation represent sickness behavior, a common final path of the sickness syndrome, which may be beneficial by promoting social immunity (362). In this model, depression is the mediator between inflammation and NTIS.

## Non-Homeostatic Mechanisms

In illness, changes of measured hormone concentration may result from exogenous factors including pharmacological effects of drugs and assay interference. They have in common that they do not represent an adaptive homeostatic reaction of the organism. As these effects are common in TACITUS, overlap with the phenotype of NTIS and can be confused with allostatic reactions, the following section delivers a short overview over triggering scenarios and their consequences.

### Drug Effects

A large number of drugs is known to influence thyroid function interfering with various mechanisms of thyroid hormone metabolism (363). Lithium and aminoglutethimide decrease thyroid hormone secretion. A high iodine load, as it ensues from amiodarone and/or radiocontrast dye application, decreases both central and peripheral deiodinases activity (364–368). In addition to causing hypodeiodination, amiodarone has antagonistic actions on T3 signaling, presumably due to its molecular similarity to thyroid hormones (369, 370). Dopamine, glucocorticoids, and somatostatin analogs suppress TSH release (371). Thyroxine absorption is altered by multiple substances including caffeine, bile acid sequestrants, sucralfate, ferrous sulfate, and aluminum hydroxide (372). This results in disruption of the enterohepatic circulation of thyroid hormones, thus contributing to reduced half-life. Moreover, many drugs alter thyroxine and triiodothyronine transport in serum such as estrogens, tamoxifen, heroin, methadone, mitotane, androgens, anabolic steroids, furosemide, NSAIDs, and salicylates by either increasing or decreasing TBG concentration or displacing them from protein-binding sites (363, 373). Antiepileptic drugs such as phenobarbital, phenytoin, and carbamazepine increase hepatic metabolism of thyroxine and triiodothyronine. In addition to amiodarone propylthiouracil, macrolides, and unselective beta-adrenergic blockers inhibit the activity of type 1 deiodinase (374), while sorafenib is able to increase D3 activity (375).

### Assay Interferences

Significant problems in FT4 and FT3 assay interpretation can arise in the varying performance of tests from different manufacturing sources (376, 377). For example many tests are affected by residual interference from effects of albumin concentrations that are often considerably reduced in NTIS and thereby lead to artificially low estimates of FT3 or FT4. In addition, some tests are insufficiently robust to the lower concentrations of thyroid hormone-binding proteins overall found in these conditions. Some drugs used in severe illness can also distort the results directly by displacing bound thyroid hormones. FT3 tests appear to suffer more in this regard than FT4 tests, though both can be compromised (377).

This can significantly affect conclusions as to the exact magnitude of allostatic effects on thyroid function and may hinder comparison of any findings from studies using different methods of measurement. The most convincing results will therefore be found using assays with minimal interference and closest adherence to the Mass Action criteria governing the working of such assays.

## Effects of Heterogeneous or Unknown Origin

The subsequently described influences originate from human civilization. They therefore fail to trigger a natural adaptive response of thyroid homeostasis. Since their action is complex, it is possible, however, that some of the associated mechanisms elicit an allostatic reaction by physiological mimicry of natural factors.

### Endocrine Disruptors

Multiple industrial substances in the environment are able to profoundly modify thyroid function (378–380). The mechanisms are as heterogeneous as the substances, and there is some overlap to drug effects (see above). This applies, e.g., to perchlorate, which inhibits iodine uptake into the thyroid and may therefore cause primary hypothyroidism (381, 382). Primary hypothyroidism may also result from exposure to polyhalogenated aromatic hydrocarbons (383) and bisphenol A (384). The effects of polychlorinated biphenyls (PCBs) include increased hepatic degradation of thyroid hormones and inhibition of deiodinase activity, which applies predominantly to cerebral D3 (385–387). Typical high-T3 syndromes under PCB exposure are therefore probably caused by decreased D3 activity rather than by stimulated D1 or D2 activity. Some plant-derived substances have thyromimetic effects and lead to decreased concentrations of TSH, FT4, and FT3 (388). In reality, endocrine disruptors are rarely present in isolated forms. Interactions among different disruptors are complex and may be additive, sub-additive, and super-additive, depending on the individual combination of substances and their respective concentrations (389–391). This makes the effects difficult to predict in the individual situation. Endocrine disruptors may result in clinical thyroid disease whose etiology is hard to pinpoint.

### Space Flight

Space journeys expose the human organism to multiple challenges. They include low or zero gravity, radiation, and impaired circadian rhythm. It is, therefore, not surprising, that manned space exploration is associated with multiple hormonal changes (392). Among them, alterations of the HPT axis seem to play a minor, but potentially significant role. Crewmembers of the Spacelab D-2 mission had slightly increased TSH concentration during flight, suggesting a form of subclinical hypothyroidism (393). These results are compatible to observations in rats and rhesus monkeys (394–397). The most plausible reason for slightly impaired thyroid function during spaceflight is the utilization of iodinated water (398). Iodine was used as a bactericidal agent in US spacecraft water systems until 1997, when a device was implemented in Space Shuttles (and later the International Space Station) to remove iodine from water before consumption. As a result post-flight TSH elevations are no longer observed in astronauts (399). However, regardless of iodine removal, in male astronauts TT4 concentrations and FT4 index continue to be higher after space flight and T3 concentrations are decreased after flight. Due to unknown reasons this NTIS-like pattern is observed in men only, but not in women (399).

## Methods of Assessment and Differential Diagnosis

Allostatic adaptations of the HPT axis frequently pose serious problems for diagnosis and differential diagnosis of thyroid disorders, because concentrations of both TSH and peripheral thyroid hormones may change considerably and fall widely outside their normal reference ranges. A diagnostic problem arises from the fact that reference ranges for thyroid parameters have been established for healthy non-pregnant adults under resting and fed conditions—a premise that is not invariably met, especially not in conditions accompanied by both severe thyroid dysfunction and systemic illness or pregnancy, where early diagnosis is essential. Moreover, even the uncompromised reference interval for TSH varies significantly by age, sex, hour of day, and ethnicity (400).

The endocrine features of TACITUS share overlapping elements with all of the following diseases, hypothyroidism (low-T3 concentration and, in chronic illness, reduced T4 concentration, and even elevated TSH in the recovery phase), hyperthyroidism (transiently increased FT4 levels and occasionally low TSH concentrations) and hypopituitarism (thyrotropic adaptation with low TSH levels despite low or normal FT4 concentrations) (73). This may present a huge challenge for differential diagnosis (24, 35). For instance, the combination of reduced TSH level and normal or even slightly increased FT4 concentration, which arises from markedly different half-lives of TSH and T4 in transitional periods of thyrotropic adaptation, may be confused with subclinical or overt hyperthyroidism (71). The situation is even more complex as the clinical spectrum of numerous severe diseases shares phenotypical features with hypothyroidism or thyrotoxicosis, respectively (72). As an example, sepsis is usually associated with fever and hyperdynamic circulation including tachycardia and peripheral vasodilatation, which are also characteristic for thyroid storm. Conversely, myxedema coma is marked by impaired vigilance, hypothermia, hypercapnia, and bradycardia, and it is therefore difficult to differentiate from critical non-thyroidal illnesses. Even subclinical hyperthyroidism may pose a risk to patients, especially to those, who are required to receive a high iodine load, e.g., in form of amiodarone or iodinated radiocontrast agents—procedures that are frequently necessary in the critically ill.

It is therefore both essential and difficult to distinguish TACITUS from diseases that are accompanied by decreased TSH levels, e.g., thyrotoxicosis and hypopituitarism. If FT3 and FT4 are both elevated or at least in the upper quintile of the respective reference ranges the diagnosis of thyrotoxicosis is straightforward. The differential diagnosis between TACITUS and subtle forms of subclinical hyperthyroidism or hypopituitarism may be more difficult, especially if an isolated thyrotropic dysfunction is present. A history of prior symptoms and signs of pituitary dysfunction may be helpful. It is also useful to evaluate the function of the corticotropic axis in patients with critical illness and possible TACITUS. In case of dysfunction treatment with glucocorticoids should be commenced prior to thyroid hormone substitution.

Hypothyroidism associates mostly with elevated TSH levels. Positive antithyroid antibodies support the diagnosis of Hashimoto thyroiditis, however without proving the diagnosis of hypothyroidism and in most cases with significant delay.

Thyroid dysfunction induced by amiodarone therapy may lead to laboratory findings similar to TACITUS. Medical and drug history may help to approach the right diagnosis. Differential diagnosis is more complex, if typical amiodarone-induced changes of thyroid function are to be distinguished from amiodarone-induced thyrotoxicosis (e.g., in tachyarrhythmia), since in both cases FT4 concentrations may be elevated (by impaired deiodination under amiodarone or by hypersecretion of thyroxine, respectively).

Today, most intensive care units are equipped with ultrasound devices. Therefore, thyroid ultrasonography may be used as an inexpensive and non-invasive method to visualize size and internal structure of the thyroid gland. Thyroid enlargement, the presence of nodules (especially of TIRADS class 2 and colloid types 2 and 3) or diffuse hyperperfusion of the thyroid are indicative of hyperthyroidism.

Challenges in the assessment of thyroid function during pregnancy result from the normal gestational changes in thyroid activity and increased prevalence of conditions that cause hyperthyroidism in pregnancy (401). Additional uncertainty arises from an ongoing controversy, if levels of total (264) or free thyroid hormones (401, 402), measured either *via* immunoassays (403) or LC/tandem mass spectrometry (404), are the preferred targets for diagnostic interpretation (248, 405). There is no doubt, however, that laboratory investigations must always be accompanied by careful clinical evaluation of the patient’s symptoms and history (401). Some professional guidelines suggested trimester-specific reference intervals for the concentrations of TSH and peripheral thyroid hormones (248, 264, 406), while the recent guideline of the American Thyroid Association recommended elevating the upper limit of the reference range back to 4 mIU/L (264). Considering the significant differences among guidelines and the conflicting results of clinical trials critical questions still remain unanswered.

Calculating model-based structure parameters of thyroid homeostasis may be helpful in differential diagnosis (407). Recent studies have used mathematical models for diagnosis and prognosis of thyroid disorders, such as Hashimoto thyroiditis and Graves’ disease (408, 409). Multiple studies showed total step-up deiodinase activity (SPINA-GD) to be significantly reduced in subjects affected by NTIS (61, 299–301). In patients with heart disease, SPINA-GD negatively correlated to age, atrial conduction time, and concentrations of B-type natriuretic peptide as well as 3,5-diiodothyronine (3,5-T2), and it predicted atrial fibrillation after cardiac surgery (61). In a study with 219 obese patients SPINA-GT, an estimate of thyroid’s secretory capacity, assisted in identifying subjects with mild secretory insufficiency of the thyroid (410). In chronic renal failure, SPINA-GT correlated to creatinine clearance, suggesting toxic effects of azotemia on thyroid function (411). Calculating the ratios of total to free T4 (TT4/FT4) (300, 301) and of total to free T3 (TT3/FT3) (300) may be helpful to screen for impaired plasma protein binding of thyroid hormones in NTIS. Jostel’s TSH index, a measure for thyrotropic anterior pituitary function (412), is decreased in patients with central adaptation in TACITUS syndrome (299). Despite accumulating evidence for the use of structure parameters in NTIS, their diagnostic utility is still insufficiently evaluated, and they have not been studied in other situations of thyroid allostasis including starvation, pregnancy, and psychiatric diseases. They have emerged, however, as valuable tools for clinical research (19–22, 24, 92, 94, 407).

## Treatment of Low-T3 Syndrome in Tacitus—An Open Question

As noted above, low-T3 syndrome and other components of NTIS correlate to severity of disease and independently predict the outcome of affected patients (54, 60, 64–66, 413–416). Guided by the idea that NTIS represents a form of illness-mediated hypothyroidism, it was suggested to treat the condition with levothyroxine (L-T4) or liothyronine (L-T3) with the expectation to improve the prognosis of critically ill patients (39, 70).

In fact certain surrogate markers, e.g., hemodynamic parameters and other markers of cardiovascular function, were demonstrated to improve after initiation of treatment with L-T3 (417, 418). With one exception of preterm infants (419) hard endpoints including survival could not be ameliorated by thyroid hormone administration (25, 170, 420, 421). In contrary, some studies observed even detrimental effects of therapy (25).

IL6-induced oxidative stress decreases the catalytic activity of D1 and D2. Selenium supplementation failed to demonstrate a beneficial effect on NTIS, although it improves critical intracellular antioxidant functions, particularly of selenoproteins (115). By providing bacteriotoxic iodine atoms, increased step-down deiodination due to stimulated D3 activity might be beneficial by defending against bacterial infections (55).

Several studies have demonstrated that treatment of the underlying disease can aid in the resolution of NTIS (422, 423).

In conclusion, universal substitution therapy cannot currently be recommended in TACITUS (424). Vastly insufficient diagnostic methods, as noted above, hinder the development of valid laboratory-based decision criteria that would help to safely identify critically ill patients with mild hypothyroidism. Diagnosis of myxedema coma therefore still relies on score systems, which either incorporate TSH and FT4 concentrations among other parameters (425, 426) or completely renounce the use of hormone measurements (426, 427).

## Conclusion

The hypothalamus–pituitary–thyroid feedback control mechanism is a dynamic adaptive system. In resting equilibrium conditions of healthy adults the behavior of all elements involved is sufficiently stable to be diagnostically interpretable. This changes dramatically in straining situations such as starvation, exhaustion, or non-thyroidal illness. In the latter situations requirements of energy, oxygen or glutathione exceed supply, prompting the control loop to switch to a different operating mode that helps to adjust consumption to available resources. This type of allostatic response is termed type 1 and it is marked by low-T3 syndrome, reduced plasma protein binding of thyroid hormones, thyrotropic adaptation, and high concentrations of rT3. A similar constellation is observed during the fetal life. An inverted response pattern is seen in cases of predictive adaptation marked by type 2 allostatic load (including obesity, endurance training, adaptation to cold and post-traumatic stress disease). Here, concentrations of TSH, FT3, and TT3 are elevated, as is the plasma protein binding of thyroid hormones. A partly similar pattern to type 2 allostasis is seen in pregnancy.

Is thyroid allostasis beneficial or harmful? Perhaps an answer can be found in the very extremes of thyroid function, thyroid storm and myxedema coma. Although both diseases are located in opposing edges of the functional spectrum, they share a special kind of interaction between dysregulation of thyroid homeostasis and increased sensitivity of the organism to altered thyroid hormone signaling. They also have a pathophysiological pattern in common: the preexisting thyroid dysfunction may remain oligosymptomatic and undiscovered for years, yet an unspecific trigger (e.g., infection, apoplexy, or myocardial infarction) may ignite a complex causal network that results in a life-threatening crisis (72, 426). Thyroid storm is a form of insufficient adaptation, since thyrotoxicosis prevents the development of TACITUS in cases of critical illness. On the other hand, myxedema coma represents a form of overcompensation, which is marked by a massive amplification of hypothyroidism by the development of NTIS in severe disease. Both forms of thyroid crisis are life threatening: thyroid storm by insufficient allostasis and myxedema coma by allostatic overload. These examples illustrate the Janus-faced character of allostasis: although lifesaving in many cases it may occasionally threaten survival through the burden of allostatic load.

Differential diagnosis of type 1 and type 2 allostasis from peripheral or central thyroid dysfunction may be difficult, the more as physiological reactions of the feedback loop overlap with non-homeostatic mechanisms including drug effects, pre-analytical factors and assay flaws in critical illness (Figure 1).

In an allostatic context correct interpretation of thyroid function cannot be based on simple diagnostic rules or laboratory tests. Rather it requires a deep understanding of physiology and a comprehensive diagnostic strategy that integrates the patient’s history, clinical parameters, and laboratory findings. Developing and validating reliable algorithms that support the required level of integration is a fundamental task for future thyroid research.

## Author Contributions

AC, AA, JD, RH, and JM made the literature research and wrote the manuscript. BD wrote the part of the manuscript thyroid in psychiatric diseases. AU wrote the part of manuscript concerning thyroid and amiodarone. HK and SH critically read and revised the manuscript. All the authors have read and approved the manuscript.

## Conflict of Interest Statement

JD received funding and personal fees by Sanofi-Henning, Hexal AG, Bristol-Myers Squibb, and Pfizer and is co-owner of the intellectual property rights for the patent “System and Method for Deriving Parameters for Homeostatic Feedback Control of an Individual” (Singapore Institute for Clinical Sciences, Biomedical Sciences Institutes, Application Number 201208940-5, WIPO number WO/2014/088516). All other authors declare that there is no conflict of interest that could be perceived as prejudicing the impartiality of the research reported.
